# Ultrastable metallic glasses formed on cold substrates

**DOI:** 10.1038/s41467-018-03656-4

**Published:** 2018-04-11

**Authors:** P. Luo, C. R. Cao, F. Zhu, Y. M. Lv, Y. H. Liu, P. Wen, H. Y. Bai, G. Vaughan, M. di Michiel, B. Ruta, W. H. Wang

**Affiliations:** 1 Institute of Physics, Chinese Academy of Sciences, 100190 Beijing, China; 2 University of Chinese Academy of Sciences, 100049 Beijing, China; 3 State Key Laboratory of Metal Matrix Composites, School of Materials Science and Engineering, Shanghai Jiao Tong University, 200030 Shanghai, China; 4 ESRF-The European Synchrotron, CS40220, 38043 Grenoble, France; 5 Univ Lyon, Université Claude Bernard Lyon 1, CNRS, Institut Lumière Matière, 69622 Villeurbanne, France

## Abstract

Vitrification from physical vapor deposition is known to be an efficient way for tuning the kinetic and thermodynamic stability of glasses and significantly improve their properties. There is a general consensus that preparing stable glasses requires the use of high substrate temperatures close to the glass transition one, *T*_g_. Here, we challenge this empirical rule by showing the formation of Zr-based ultrastable metallic glasses (MGs) at room temperature, i.e., with a substrate temperature of only 0.43*T*_g_. By carefully controlling the deposition rate, we can improve the stability of the obtained glasses to higher values. In contrast to conventional quenched glasses, the ultrastable MGs exhibit a large increase of *T*_g_ of ∼60 K, stronger resistance against crystallization, and more homogeneous structure with less order at longer distances. Our study circumvents the limitation of substrate temperature for developing ultrastable glasses, and provides deeper insight into glasses stability and their surface dynamics.

## Abstract (web summary)

Producing ultrastable metallic glasses has always been associated with substrates heated close to the glass transition temperature. Here, the authors show that reducing the deposition rate of the metallic glass on a cold substrate produces ultrastable metallic glasses with remarkably improved stability.

## Introduction

Glasses are generally produced by cooling a liquid very rapidly to circumvent crystallization^1,2^. The glass transition is a kinetic process^1,2^, and the slower is the cooling rate, the larger is the time available for the supercooled liquid to rearrange its structure and follow the temperature changes before to freeze in the glassy state. Following this observation, one could think to tune the properties of a glass by simply controlling the rate of cooling. However, the effect of the cooling rate is quite small, as one order of magnitude faster rate merely increases the glass transition temperature, *T*_g_, by ≈3 K in both polymers^3^ and metallic glasses (MGs)^4^. This increment is definitely too small for the majority of technological purposes.

In the past decade, Ediger and co-workers demonstrated that by virtue of physical vapor deposition the kinetic limitations in traditional glass formations can be bypassed, and it is possible to synthesize organic molecular glasses with extraordinary thermodynamic and kinetic stability^5^, and exceptional mechanical properties^6^. These glasses, known as ultrastable glasses, exhibit much higher *T*_g_ with respect to their ordinary counterparts produced by conventional liquid cooling. Similar properties would be expected from liquid-cooled glasses only after aging for thousands of years or more^7,8^. In subsequent efforts, more species of ultrastable glasses, such as MGs^9,10^ were fabricated by vapor deposition, and it has been possible to obtain even Lennard-Jones ultrastable glasses^11^ by numerical simulations. In all these works, the general consensus is that the substrate temperature (*T*_sub_) during deposition is a critical variable, and that the optimal *T*_sub_ for creating ultrastable glasses is near the *T*_g_ of ordinary glasses, e.g., *T*_sub_ = 0.8 ∼ 0.9*T*_g_ (refs. ^5–12^).

It has been demonstrated in various glasses that the surface mobility is several orders of magnitude faster and exhibits weaker temperature dependence than that of the bulk^13–18^. This enhanced surface mobility is thought to play a key role in the formation of ultrastable glasses via vapor deposition^5–12^. By increasing *T*_sub_, the mobility of atoms or molecules at the surface is significantly enhanced. This allows the constituents to sufficiently reorganize and explore stable configurations, resulting then in the formation of ultrastable glasses^5–12^. Although the creation of ultrastable polymer glasses by the assembly of polymer nanoglobules is different from typical vapor deposition, a high *T*_sub_ ≈ 0.87*T*_g_ is still required^19^. The coincidence of the optimal *T*_sub_ for preparing ultrastable glasses with the ideal glass transition temperature (Kauzmann temperature) has also led to the argument that there is an underlying thermodynamic mechanism governing the formation of ultrastable glasses^11^. Following all these works^5–12,19^, high *T*_sub_ near *T*_g_ has been considered as a prerequisite in synthesizing ultrastable glasses, while low *T*_sub_ has been suggested to decrease both the thermodynamic role and the surface dynamics^12^.

Here, we contradict this empirical rule by showing that it is possible to obtain ultrastable MGs on cold substrate with *T*_sub_ far below *T*_g_ (*T*_sub_ ≈ 0.43*T*_g_). Substantial enhancement of glass stability can be achieved by lowering the deposition rate, *R* to ∼1 nm min^−1^. In comparison with ordinary glasses obtained by liquid quenching, our glasses exhibit much higher stability with an increase of *T*_g_ up to ∼60 K, a more homogeneous structure, and a stronger resistance to crystallization. More importantly, our work suggests that the relaxation dynamics at the surface of the glass could be much faster-than-expected despite of the low temperature substrate, and call for a reconsideration of the prerequisites for ultrastability of glasses.

## Results

### Experiment

A Zr_46_Cu_46_Al_8_ (at.%) target was utilized for ion beam assisted deposition (IBAD). Different from previous works, we leave the substrate at room temperature which corresponds to *T*_sub_ ≈ 0.43*T*_g_. Details about the sample preparation and subsequent measurements can be found in the Methods section. Chemical analysis (Thermo IRIS Intrepid II XSP) confirmed that the compositions of the vapor-deposited glass films are identical to that of the ordinary glass ribbon prepared by melt-spinning method within 1% error (Supplementary Table 1). This is further ascertained by their similar melting temperatures (Supplementary Figure 1). No detectable oxygen in the deposited films was found, as confirmed by an oxygen and nitrogen analyzer. The deposited MG films have atomic level smooth and homogeneous surface structure with a root-mean-square surface roughness ≈0.1 nm (Supplementary Figure 2).

### Calorimetric analyses

**Fig. 1:**
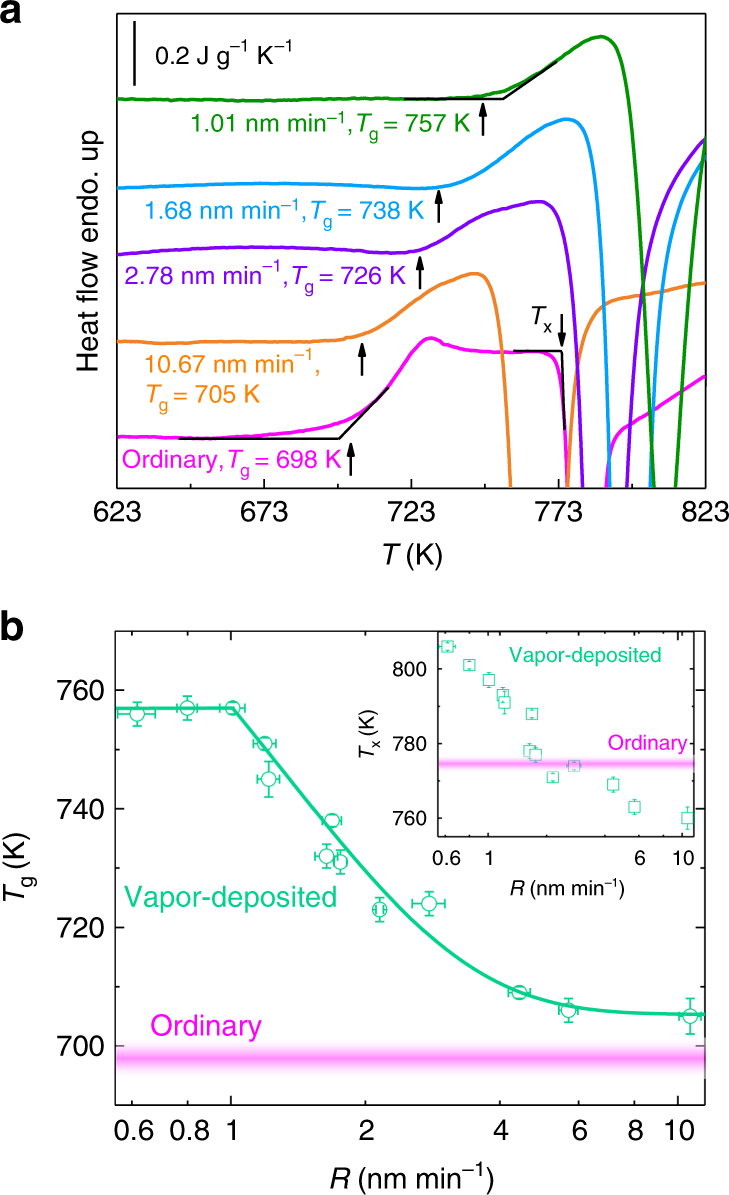
Effect of vitrification routes on the glass transition and crystallization. **a** Representative DSC traces at a heating rate of 20 K min^−1^ for Zr_46_Cu_46_Al_8_ MGs: ordinary glass produced by melt-spinning technic; vapor-deposited glass films at different rates as denoted below each curve. *T*_g_ and *T*_x_ are defined from the onset of the transformation as indicated by the intersection of the black lines. **b** *T*_g_ vs. *R*. The solid line at high *R* is an exponential fit to the data, at low *R* the *T*_g_ keeps invariant. Inset: *T*_x_ vs. *R*. The error bars indicate the standard deviation of three to five measurements. For comparison, the *T*_g_ and *T*_x_ with their variation ranges for the ordinary glass are presented by the shaded magenta areas

Figure 1a shows the differential scanning calorimetry (DSC) profiles of Zr_46_Cu_46_Al_8_ MG obtained with different preparation routes. The magenta curve is for the ordinary glass prepared by liquid quenching, and its *T*_g_ is 698 K consistent with previous reports^20^. The other DSC curves are for glass films synthesized by vapor deposition at different rates. As can be seen, *T*_g_ increases with decreasing deposition rate. When the deposition rate is 10.67 nm min^−1^, the glass film has a similar *T*_g_ (705 K) to that of the ribbon. As the deposition rate is lowered to 1.01 nm min^−1^, the *T*_g_ of the corresponding glass increases to 757 K, which is 59 K higher than that of the ordinary glass. This 8.5% increase of *T*_g_ at *T*_sub_ ≈ 0.43*T*_g_ is even larger than the previously reported value (7.1%) at high *T*_sub_ ≈ 0.8*T*_g_ for a Zr_55_Cu_30_Ni_5_Al_10_ MG (ref. ^10^). At the same *T*_sub_ of 0.8*T*_g_, Yu et al. reported instead an increase of *T*_g_ by only 1.6% for a Zr_65_Cu_27.5_Al_7.5_ MG^9^. This difference with the pioneering work of Yu et al.^9^ is likely due to the employed deposition rate of 84 nm min^−1^, much higher than that used in ref. ^10^ (11.4 nm min^−1^) and in our study. These comparisons highlight thus the importance of the deposition rate in improving the stability of the deposited glass.

Figure 1b shows the semi-log plot of *T*_g_ vs. *R* for the vapor-deposited glasses, together with the *T*_g_ of the ordinary ribbon (shaded magenta area). For *R* < 1.01 nm min^−1^, *T*_g_ appears constant around 757 K. This plateau at low *R* sets a lower limit on the experimentally meaningful deposition rate, as further decreasing *R* does not lead to higher *T*_g_ within the experimental sensitivity. When *R* is larger than 1.01 nm min^−1^, *T*_g_ decreases exponentially with increasing *R*, and at *R* ≈ 10 nm min^−1^, *T*_g_ gradually approaches the value of the ribbon. It is clear that the kinetic stability as quantified by the value of *T*_g_ is strongly influenced by the rate of deposition: lowering the deposition rate by merely one order of magnitude leads to significantly enhanced *T*_g_ by as large as 60 K. Interestingly, *T*_x_ is also affected by the deposition rate. This is shown in the inset in Fig. 1b where the onset temperature of crystallization, *T*_x_, vs. *R* is presented. Different from the non-monotonic variation of *T*_g_, *T*_x_ increases monotonically with decreasing the deposition rate. For *R* > 2 nm min^−1^, the *T*_x_ of the vapor-deposited glasses is even lower than that of the ordinary glass. For lower *R*, the increased *T*_x_ suggests the higher stability against crystallization of the ultrastable glasses.

### Surface mobility

The formation of ultrastable MGs at *T*_sub_ far below *T*_g_ suggests that the atoms at the surface still retain sufficient mobility even at lower temperatures. The slow rate of deposition makes the atoms have enough time to rearrange on the surface, and the system can explore more stable configurations before buried by the upcoming atoms. When the deposition rate is lower than 1.01 nm min^−1^, the value of *T*_g_ reaches a plateau, suggesting that the timescale set by this critical rate is close to that of the surface dynamics. The free surface residence time^21^ before a fresh atomic layer is deposited can be estimated by dividing the thickness of one monolayer by the deposition rate. The atomic weighted diameter, as calculated by weighting the atomic diameter of each component to their composition, can be taken as a measure of the thickness of the top monolayer. Given the atomic radius of 0.160 nm for Zr, 0.128 nm for Cu, and 0.143 nm for Al, the thickness of the surface monolayer is estimated to be ≈0.288 nm. At the deposition rate of 1.01 nm min^−1^, the free surface residence time is therefore estimated to be ≈17 s. Based on the fact that further lowering the deposition rate will not affect the *T*_g_ value (Fig. 1b), it is reasonable to conclude that 17 s is long enough for atoms at the free surface to rest and freely explore stable configurations before buried. Successive layer by layer deposition results thus in a glass with highly enhanced stability. Following this argument, the timescale associated with the surface dynamics should be close to 17 s as well.

### Crystallization upon annealing

**Fig. 2:**
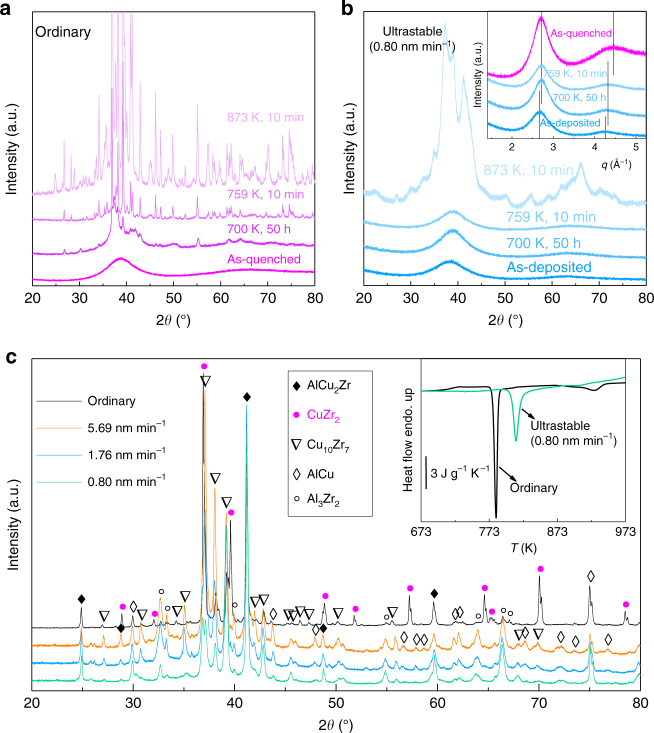
Crystallization dynamics and phase formation of the ordinary and the ultrastable glasses upon annealing. **a**, **b** XRD profiles for as-prepared and annealed **a** ordinary and **b** 0.80 nm min^−1^ deposited ultrastable MGs. The annealing temperature and time are denoted by the legend values above each curve. Inset in **b**: XRD data for as-deposited and annealed ultrastable MGs and as-quenched ordinary MGs plotted as a function of *q* = 4*π*sin*θ*/*λ*. The vertical lines indicate the peak positions. **c** XRD profiles for samples after annealed at 973 K for 1 h, from top to bottom, the ordinary, 5.69 nm min^−1^, 1.76 nm min^−1^, and 0.80 nm min^−1^ vapor-deposited glasses. Inset: DSC traces for the ordinary and the 0.80 nm min^−1^ deposited glasses at a heating rate of 20 K min^−1^

The significantly improved stability of our ultrastable glasses is further confirmed by studies on the kinetics of the structural evolution under isothermal annealing. By means of laboratory X-ray diffraction (XRD), we characterized the structure of both ordinary and ultrastable MGs heated at 20 K min^−1^ to different annealing temperatures. Figure 2a, b shows, respectively, the XRD patterns for the ordinary and the ultrastable MGs, in the as-prepared and annealed states. The presence of a unique broad diffraction peak in the as-prepared samples indicate their fully amorphous nature. After annealing at 700 K for 50 h, and at 759 K and 873 K for 10 min, sharp Bragg peaks emerge in the XRD spectra of the ordinary glass, indicating the precipitation of crystalline phases (Fig. 2a). In contrast, the ultrastable glasses are more resistant against crystallization (Fig. 2b). They remain still completely amorphous after annealing at 700 K and 759 K. To trigger the crystallization, it is necessary to anneal the glass at much higher temperature (873 K). In this case, DSC measurements indicate that crystallization in the ultrastable glasses has been completed (inset in Fig. 2c). In contrast to the ordinary glass, no well-defined Bragg peaks show up in the ultrastable glasses during this annealing, suggesting that the precipitated phases are smaller in size and not well ordered in structure. These observations demonstrate the distinctly different crystallization process in our ultrastable glasses. While crystallization in the ordinary glass occurs through fast nucleation and grain growth, our results suggest that there are probably more nucleation sites in the ultrastable glasses, but the grain growth is very slow. This hypothesis is supported by DSC measurements (inset in Fig. 2c). The crystallization process in the ordinary glass covers a range of ≈14 K with a sharp exothermal peak, and it takes only ≈0.7 min to complete at the heating rate of 20 K min^−1^. For the ultrastable glass, the crystallization initiates, however, at much higher temperature and covers a broader range of ≈27 K. These results confirm the high kinetic stability of the glasses formed at slow deposition rate, in agreement with their enhanced *T*_g_.

Furthermore, the precipitated crystalline phases in the ultrastable glasses are found to be different from that in the ordinary glass, strengthening the idea of the presence of different local atomic configurations. To compare the phase formation, the glasses were annealed at 973 K for 1 h. The annealing temperature is below the onset temperature of the B2 phase transition in this alloy^22^, so that the possible effect of crystalline phase transition can be excluded. Figure 2c displays the XRD patterns for the annealed ordinary and vapor-deposited glasses. In the annealed ribbon, the precipitated phases are identified to be mainly intermetallic AlCu_2_Zr and CuZr_2_ with minor amount of AlCu, Cu_10_Zr_7_, and Al_3_Zr_2_. Differently, the XRD patterns for the annealed ultrastable glass does not show the presence of CuZr_2_ phase, and the primary phases in the ultrastable glasses are AlCu_2_Zr and Cu_10_Zr_7_. This indicates that the precipitation of CuZr_2_ is suppressed in the ultrastable glasses. The patterns for the vapor-deposited glasses are very similar, but the peak intensity of Cu_10_Zr_7_, AlCu, and Al_3_Zr_2_ phases increases as the deposition rate is increased, indicating the effect of deposition rate on the formation of crystalline phases.

### Structure characterization

**Fig. 3:**
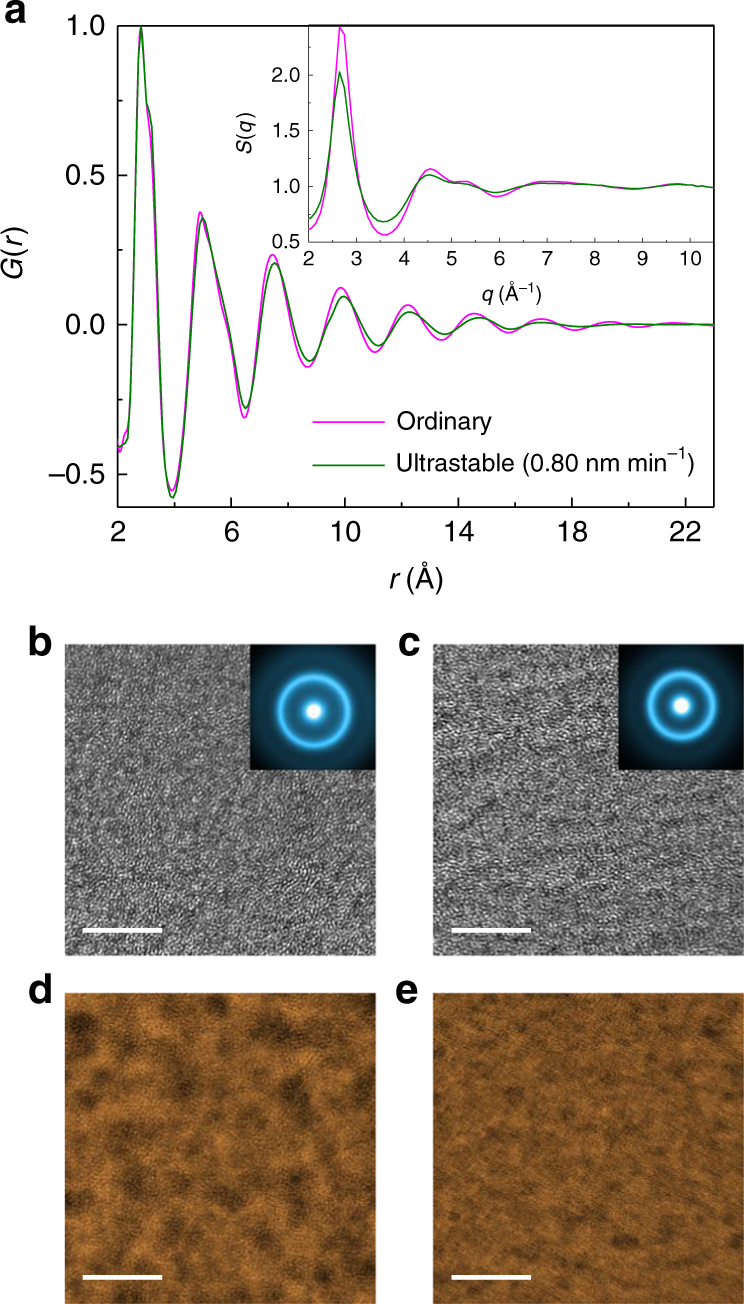
Structure characterization for the ordinary and the 0.80 nm min^−1^ deposited ultrastable glasses. **a** Pair distribution function *G*(*r*) and static structure factor *S*(*q*) (the inset) for the ordinary and the ultrastable MGs. **b**, **c** HRTEM images of **b** the ordinary and **c** the ultrastable MGs. Insets: selected area electron diffraction (SAED) patterns taken from a large selected area. **d**,** e** HADDF-STEM images of **d** the ordinary and **e** the ultrastable MGs. The scale bars in **b**–**e** are 5 nm

The different crystallization dynamics and phase precipitation between the ultrastable and the ordinary glass indicate that substantial structural changes have been imparted to the glass during the deposition. Indeed, a closer look at Fig. 2a, b shows that the two as-prepared glasses do not have the same structure. The inset in Fig. 2b shows the XRD data for the as-deposited and annealed ultrastable MGs and the as-quenched ordinary MGs as a function of the wave vector *q*, being *q* = 4*π*sin*θ*/*λ*, and *θ* and *λ* the scattering angle and the wavelength of the incident beam, respectively. With respect to the ordinary glass, the ultrastable glass shows less intense and broader peaks, slightly shifted toward lower *q* values. These structural differences are confirmed also by high-energy synchrotron XRD measurements (see the static structure factor *S*(*q*) in the inset in Fig. 3a) and correspond to changes in the local atomic structure. These changes can be better appreciated by real space analysis, through the evaluation of the pair distribution functions, *G*(*r*). As shown in Fig. 3a, the *G*(*r*) of the ordinary glass presents well-defined oscillations up to the 8th neighboring shell, while the ultrastable glass is more glassy and exhibits an ordered structure only up to the 6th shell. This means that the ordinary glass is more ordered at longer distances, and is characterized by more packed shells, as suggested by the shifts of the peaks of the *G*(*r*) toward shorter distances. Interestingly, structural modifications have been reported also for organic glasses produced by vapor deposition at high *T*_sub_ (refs. ^23,27^).

Further structural characterizations have been performed also by using transmission electron microscopy (TEM). The high-resolution TEM (HRTEM) images appear to be similar for both ordinary (Fig. 3b) and ultrastable glass (Fig. 3c), such as the homogeneous maze-like patterns. Differently, scanning transmission electron microscopy (STEM) is more sensitive to the local structure and can project the local atomic arrangements in real space. Large variations in contrast can be observed in the ordinary glass by high-angle annular dark-field scanning TEM (HADDF-STEM) (Fig. 3d), indicating its heterogeneous atomic arrangements^28^. On the contrary, the contrast variation is much lower in the ultrastable glass (Fig. 3e), indicative of a more homogeneous atomic structure. Since all TEM specimens are prepared by ion milling under the same conditions, such difference between the ultrastable and the ordinary glass can be attributed to their distinct structural packing and configuration, in agreement with the XRD results. In molecular systems, the enhanced stability can be correlated with molecular orientation or layered packings^25,26,29^. Differently, MGs are made of isotropic constituents, ruling out the possible effect from molecular orientation. However, as a multi-component material, further investigations are warranted to distinguish the role of the local atomic structure and layered packing in the increased stability of MG, which is crucial for clarifying the process of producing stable MG.

## Discussion

**Fig. 4:**
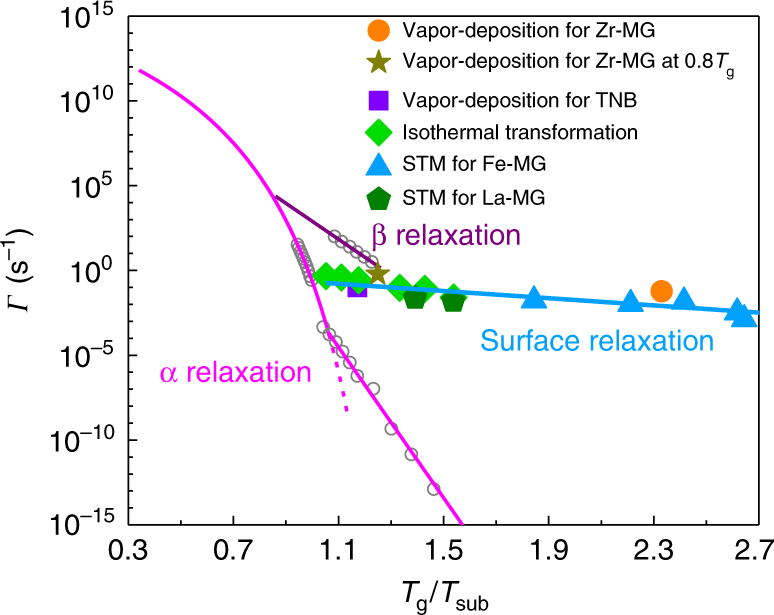
Comparison of surface relaxation with bulk α and β relaxations. The magenta and purple curves along with the open circles show the bulk α and β relaxation rates vs. *T*_g_/*T* in MGs^35^, respectively. The solid square, star, and circle show the surface relaxation rate *Γ*_surface_ vs. *T*_g_/*T*_sub_ estimated from vapor deposition of an ultrastable molecular glass^5^, the Zr-MG of ref. ^10^, and the sample studied in the present work, respectively. The rhombuses are for ethylcyclohexane glass as *Γ*_surface_ is the inverse critical free surface residence time at which the time of isothermal transformation from the glass into the supercooled liquid state reaches the plateau (Figure 9 in ref. ^21^). The *Γ*_surface_ from STM measurements are plotted as pentagons (left one: La_50_Ni_15_Al_25_Cu_10_, right one: La_60_Ni_15_Al_25_ MGs^32^) and as triangles (Fe_78_B_13_Si_9_ MG^33, 34^). The blue line is a linear fit to the surface relaxation rates

The unique deposition rate dependence of *T*_g_ shown in Fig. 1b sheds additional light on the surface properties of MGs, especially at such a low *T*_sub_. The increasing stability with decreasing deposition rate supports the idea of an enhanced surface mobility mechanism, analogous to the one occurring at high *T*_sub_^7,21^. In addition, the fact that just one order of magnitude lower rate of deposition generates a huge enhancement in glass stability even at a *T*_sub_ far below the Kauzmann temperature^30^ (Fig. 1b), questions the argument of an underlying thermodynamic mechanism governing the formation of stable glasses^11^. In numerical simulations^31^, it was found that the optimal *T*_sub_ for a given deposition rate decreases as deposition slows, suggesting a competition between thermodynamics and kinetics. This study shows that the stability keeps increasing monotonically even after four orders of magnitude decrease of the deposition rate^31^. In contrast, experiments on an ethylcyclohexane glass prepared at *T*_sub_ = 0.6 ∼ 0.95*T*_g_ (ref. ^21^) and our MGs obtained at *T*_sub_ = 0.43*T*_g_ show the occurrence of a lower limit in the increasing of the glass stability within just one to two orders of magnitude slower deposition rates. These discrepancies are likely to be a consequence of the difference in procedures for vapor deposition employed in numerical simulations and experiments, and highlight the importance of kinetics during stable glass formation with much faster-than-expected surface dynamics. From the preparation of ultrastable molecular glasses, Ediger et al. estimated a relaxation time of 10 s for the top surface layer at *T*_sub_ = 0.85*T*_g_ (ref. ^5^). In our work, the deposition rate has been lowered to the critical limit of ≈1 nm min^−1^ below which *T*_g_ remains unaffected. The estimated surface relaxation time of ≈17 s is in good agreement with the direct probing of atomic rearrangements at the surface of MGs obtained by scanning tunneling microscope (STM) measurements^32–34^. This is shown in Fig. 4 where we report an Arrhenius diagram displaying the surface relaxation rates (*Γ*_surface_, inverse relaxation time) as a function of *T*_g_/*T*_sub_ for different glasses and measured by different approaches. The data for bulk α and β relaxations of MGs measured in our previous study^35^ are shown for comparisons, where the bulk α relaxation follows a Vogel–Fulcher–Tammann (VFT) fashion in supercooled liquid state and an Arrhenius fashion in glassy state. Despite of the diverse nature of the systems and experimental techniques used for estimating the surface relaxation rates, the data collapse on a single curve which can be described by an Arrhenius process with an activation energy of 2.4(±0.4) *k*_B_*T*_g_. This value is much smaller than that reported for the bulk α (≈52*k*_B_*T*_g_) and β (≈26*k*_B_*T*_g_) relaxations of different MGs^35^. As a further confirmation of our estimation, we have also calculated the surface dynamics for the Zr-based glass film reported in ref. ^10^ prepared at *T*_sub_ = 0.8*T*_g_ and with a lower deposition rate than ref. ^9^ (the star in Fig. 4). This value also collapses into the surface relaxation curve. Upon further lowering of the deposition rate, higher stability may most likely be obtained, leading to a lower value and thus an even better agreement.

Figure 4 provides a simple but ample look of the surface relaxation dynamics in glass formers, which is still relevant to stable glass formation even at low *T*_sub_. In stark contrast to the β relaxation^36,37^ and the α relaxation^35,38^ occurring in the bulk, the temperature dependence of surface relaxation is almost negligible, analogous to a recently discovered fast mode likely associated to local stress dipoles^35^. Although the nature of the surface relaxation cannot be elucidated within this study, our results suggest that even at low substrate temperatures, atomic motions at free surface remain still active and are many orders of magnitude faster than the structural rearrangements in the bulk, as shown in Fig. 4. Interestingly, previous measurements have shown that the surface relaxation time of glassy polymers exhibits no discernible temperature dependence when going somewhat below *T*_g_ (ref. ^13^). Recently, it was found in ultrathin films of a molecular glass that the activation energy of the average film dynamics decreases dramatically with decreasing film thickness and eventually becomes even much smaller than that of the surface diffusion, suggesting as well the very weak temperature dependence of surface relaxation^18^. These findings agree with our suggestion that the dynamics at the glass surface is much faster than expected, even at low temperatures. A confirmation of this hypothesis will however require direct measurements of free surface relaxation dynamics in several glass formers. As multicomponent systems, it is likely that each element in MGs moves with a different timescale^39^, even if STM measurements^32–34^ suggest more cooperative rearrangements at the surface. Further investigations are necessary to clarify this issue, as the knowledge of the mobility of each constituent element is important to better understand the surface mobility and the ultrastability.

Finally, it is worth stressing that the successful preparation of MGs with much improved stability at room temperature is of technological significance. MGs are regarded as extraordinary materials possessing unique mechanical and functional properties^40,41^. The use of high *T*_sub_ during deposition brings additional technical problems as most MGs can only be deposited on substrates with high temperature resistance like NaCl^9^ or Si^10^. Consequently, their applications will be severely restricted if the substrate cannot stand high temperatures. This would be the case, for instance, for wear and corrosion resistant coating^42^, or in their use as components of composite materials. Our work not only provides access to ultrastable MGs at *T*_sub_ far below *T*_g_, but also shows the experimental route necessary for obtaining even better stability, which will enable additional possibilities for wider technological applications.

In summary, we show the production of ultrastable MGs with increased stability by keeping *T*_sub_ at room temperature (≈0.43*T*_g_ for Zr-based MG), questioning therefore the general empiric rule of thumb of keeping the substrate at 0.8 ∼ 0.9*T*_g_ (refs. ^5–12,19,21^). By lowering the deposition rate to ≈1 nm min^−1^, we have been able to produce glasses with improved stability against crystallization, and with ∼60 K enhanced *T*_g_, significantly larger than that in conventional quenched glasses and in previous measured ultrastable MGs^9,10^. Intriguingly, the stability improves on lowering the deposition rate until a low critical limit of ≈1 nm min^−1^. An estimation of the timescale associated to the surface dynamics provides a characteristic time of ≈17 s, in good agreement with the data measured with STM in other MG systems. Structural analyses show that the ultrastable glasses are characterized by a more homogenous structure, with less order at larger distances, more stability and different phase formations during crystallization. The formation of stable glasses at *T*_sub_ far below *T*_g_ will not only boost the scientific research on ultrastable glasses and surface relaxation, but may also allow defining a route to improve coatings on materials and design better amorphous alloys.

## Methods

### Metallic glasses preparation

Alloys with a normal composition of Zr_46_Cu_46_Al_8_ (at.%) were prepared by arc-melting pure metals in a Ti-getter high-purity argon atmosphere. MG ribbons with 20 μm thick were produced by melt-spinning technic.

### MG films prepared by IBAD

The Zr_46_Cu_46_Al_8_ deposition target with a size of 100 × 100 × 2 mm^3^ is prepared by copper-mold-casting. Such a large area ensures the full coverage of the ion beam. Preceding the deposition of the MG, one layer of aluminum with ∼100 nm thick was firstly deposited on a flat polycarbonate (PC) plate with ∼12 cm in diameter, and then the chamber is opened for about 5 min for this aluminum layer to be exposed to atmosphere to form an oxidized layer on the surface. The formation of an oxidized aluminum layer makes the following deposited MG film being more easily detached. To strike off the possibly existing oxide layer on the target surface, pre-sputtering was carried out for 200 s, then a MG film with ∼2 μm thick was deposited. By tuning the ion beam current (i.e., from 2–70 mA) we can obtain different deposition rates. The beam energy is 750 eV. The base pressure of the chamber is better than 2 × 10^−4^ Pa, the depositing argon pressure is 2.4 × 10^−2^ Pa. The as-received films were detached from the PC substrate by dissolving the in-between aluminum layer into a 1 mol L^−1^ NaOH solution, and then the impurities on the films were removed using deionized water. Effect of the substrate materials and comparison between different deposition techniques are discussed in Supplementary Note 1.

### Differential scanning calorimetry analysis

A Perkin-Elmer DSC 8000 was employed to examine the kinetic and thermodynamics of the samples at a heating rate of 20 K min^−1^ with 20 mL min^−1^ flowing pure argon gas to prevent possible surface oxidation. The mass of DSC samples is 15 ∼ 20 mg to achieve good data accuracy. For each sample, at least three measurements were performed for statistical calculation of characteristic temperatures, e.g., the onset glass transition temperature, *T*_g_, and crystallization temperature, *T*_x_.

### Near *T*_g_ annealing

The annealing was conducted in a muffle furnace with temperature stability of ±1 K. Ordinary MG ribbons and 0.80 nm min^−1^ deposited films were studied. The samples were sealed in quartz tubes with high-purity argon gas to prevent oxidation and heated to the expected annealing temperatures at a rate of 20 K min^−1^. Three annealing procedures are studied at 700 K for 50 h, at 759 K for 10 min, and at 873 K for 10 min. To fully crystallize the amorphous samples and compare their crystalline phase formation, we heated the samples at 20 K min^−1^ to 973 K and then annealed at this temperature for 1 h. The annealed samples were examined by laboratory XRD to characterize their structures.

### X-ray diffraction

The measured glass samples were put onto a zero-background silicon wafer, and laboratory XRD patterns were collected with a Bruker D8 Advance with a Cu *K*_α_ source in the Bragg–Brentano geometry. High-energy XRD measurements were carried out with an incoming beam energy of 79.5 keV at the ID15A beamline at ESRF, Grenoble (F). At 79.5 keV, the calculated X-ray attenuation for the 2 μm thick deposited glass film varies from 0.17% at *q* = 0 Å^−1^ to 0.24% at *q*_max_ = 30 Å^−1^. The calculated value for the 20 μm thick ribbon varies instead from 1.7% at *q* = 0 Å^−1^ to 2.4% at *q*_max_ = 30 Å^−1^. The attenuation is therefore linearly proportional to the sample thickness, weakly dependent on *q*, very small, and consequently negligible. The diffracted signal is also linearly proportional to the sample thickness. For such reasons, it is not necessary to use samples of similar thickness.

The incident flux normalization is done with a diode placed in front of the sample. Diffraction patterns were collected in transmission geometry by using a Pilatus3 X CdTe 2M hybrid photon counting detector. The detector was off-centered with respect to the incident beam and located close to the sample to access up to *q* ≈ 30 Å^−1^ and to be able to calculate with a good resolution the pair distribution function *G*(*r*). The intensity profiles were corrected for the background contribution, polarization of the X-rays, and detector geometrical correction. The *G*(*r*) and *S*(*q*) were calculated using routines from the Diffpy-CMI library^43^ with some local modifications for outlier rejection and treatment of background effects.

### Transmission electron microscopy

Specimens for TEM characterization were carefully prepared by ion milling with 3 keV Ar ions at the liquid nitrogen temperature. HRTEM and HAADF-STEM observations were conducted using a cold field emission TEM (JEM-ARM200F, JEOL) equipped with a spherical aberration (Cs) corrector for the probe-forming objective lens.

### Data availability

The data that support the findings of this study are available from the corresponding author upon request.

## Electronic supplementary material

Supplementary Information(PDF 352 kb)
