# Supplementary material for: Ultrastable metallic glasses formed on cold substrates
Source: Nat Commun. 2018 Apr 11;9:1389. doi: 10.1038/s41467-018-03656-4 (PMC5895802; doi:10.1038/s41467-018-03656-4)
Supplement: Supplementary file 1 — Supplementary Information(PDF 352 kb) [file 41467_2018_3656_MOESM1_ESM.pdf]

Supplementary Information for

Ultrastable metallic glasses formed on cold substrates

Luo *et al*.

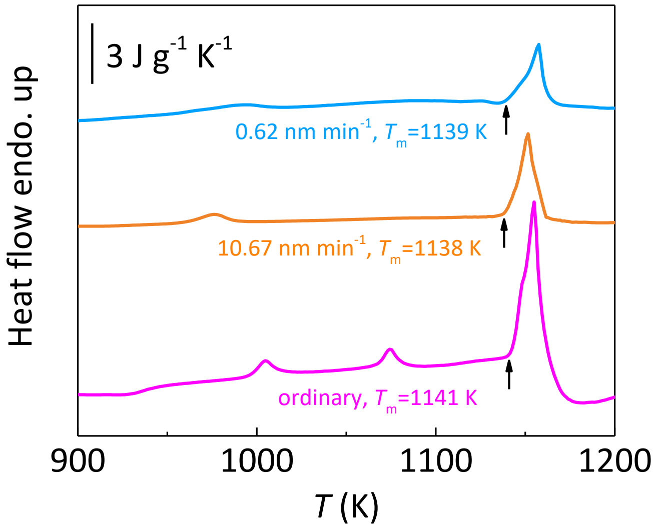

Supplementary Figure 1 The melting behavior of ordinary liquid quenched MG ribbon and vapor-deposited MG films at 10.67 nm min^{-1} and 0.62 nm min^{-1} measured using a NETZSCH DSC 404 at a heating rate of 20 K min^{-1}. The melting temperatures T_m as indicated by black arrows are similar for the samples, T_m=1141 K for ordinary liquid quenched MG ribbon, T_m=1138 K for 10.67 nm min^{-1} and 1139 K for 0.62 nm min^{-1} deposited films.

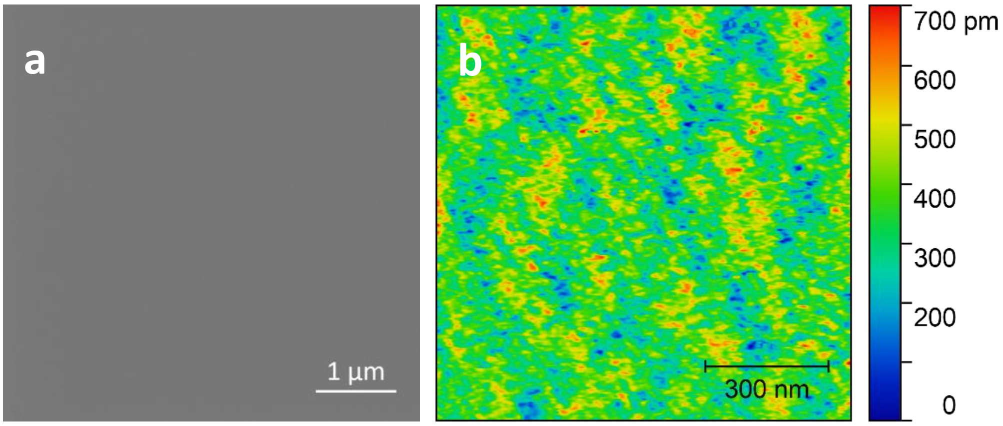

Supplementary Figure 2 Surface morphology of 0.80 nm min^{-1} deposited MG films. **a** scanning electron microscopy (SEM, Phenom XL) image. **b** atomic force microscopy (AFM, Bruker Dimension Edge) image. The root-mean-square surface roughness ≈0.1 nm, similar to that obtained by magnetron sputtering with a root-mean-square surface roughness of 0.1~ 0.3 nm (Ref. 1).

Supplementary Table 1 Compositions determined by chemical analysis (Thermo IRIS Intrepid II XSP), glass transition temperature (T_g) and crystallization temperature (T_x) (at a heating rate of 20 K min^{-1}) of liquid quenched metallic glass (MG) ribbon and vapor-deposited MG films at several representative rates *R*. The experimental sensitivity of chemical analysis is within 1%. The small amount of Hf and Fe may be induced by the impurity of raw materials or during preparation.

R (nm min^{-1})	Composition (at. %)	T_g (K)	T_x (K)
ribbon	$Zr_{45.43}Cu_{45.42}Al_{8.27}Hf_{0.49}Fe_{0.39}$	698±3	775±3
10.67±0.61	$Zr_{46.03}Cu_{45.09}Al_{8.15}Hf_{0.59}Fe_{0.14}$	705±3	760±3
5.69±0.28	$Zr_{46.47}Cu_{44.46}Al_{8.16}Hf_{0.64}Fe_{0.27}$	706±2	763±2
1.64±0.10	$Zr_{45.15}Cu_{45.88}Al_{8.18}Hf_{0.49}Fe_{0.30}$	732±2	778±2
1.22±0.07	$Zr_{45.46}Cu_{45.34}Al_{8.39}Hf_{0.54}Fe_{0.27}$	745±3	791±3
0.80±0.04	$Zr_{45.68}Cu_{45.15}Al_{8.36}Hf_{0.52}Fe_{0.29}$	757±2	801±1

Supplementary Note 1 Effect of the substrate materials and comparison between different deposition techniques.

We believe that the substrate material used during the preparation of ultrastable glasses is not important, even if we cannot exclude that is totally irrelevant. For the preparation of organic[2] and of metallic[3] glass films, the samples were deposited onto an aluminum pan for calorimetric (DSC) measurements. In our work, as shown in the Method section in the main text, we use the same substrate materials as in Refs 2 and 3. The only difference is that we deposit the sample onto an aluminum film which is firstly deposited onto a flat polycarbonate (PC) plate and removed by aqueous alkali after the sample deposition. Differently, in Ref. 4 the authors used a NaCl substrate. However, we believe that the formation of different ultrastable glasses is due to the fact that they employed a deposition rate of 84 nm min^{-1}, thus orders of magnitude higher than that used in our study.

Concerning the deposition technique, ion beam assisted deposition (IBAD) is very

similar to the magnetron sputtering employed in Refs 3 and 4, as both methods consist in sputtering techniques using usually Ar as ion source. The quality of the MG films deposited by IBAD and magnetron sputtering is very similar. For example, magnetron sputtering made smooth and homogeneous MG films with a root-mean-square surface roughness of 0.1~0.3 nm (Ref. 1) by using deposition parameters as those employed in Ref. 3 and 4. Our glass films have also a similar surface (root-mean-square surface roughness ≈0.1 nm, shown in Supplementary Figure 2). We think therefore that the improved stability of our MG films is not related to technical differences during deposition but more to the substrate temperature and the low deposition rate.

Organic glassy films where instead prepared by means of thermal evaporation deposition[2], which is a different deposition technique that cannot be used for metal materials.

Supplementary References

1. Liu, Y. H., Fujita, T., Hirata, A., Li, S., Liu, H. W., Zhang, W., Inoue, A. & Chen., M. W. Deposition of multicomponent metallic glass films by single-target magnetron sputtering. *Intermetallics* **21**, 105–114 (2012).
2. Swallen, S. F., Kearns, K. L., Mapes, M. K., Kim, Y. S., McMahon, R. J., Ediger, M. D., Wu, T., Yu, L. & Satija, S. Organic glasses with exceptional thermodynamic and kinetic stability. *Science* **315**, 353–356 (2007).
3. Aji, D. P. *et al*. Ultrastrong and ultrastable metallic glass. Preprint at arXiv:1306.1575 (2013).
4. Yu, H. B., Luo, Y. & Samwer, K. Ultrastable metallic glass. *Adv. Mater.* **25**, 5904–5908 (2013).
